# The effects of prostate volume and PI-RADS category on optimal PSA-density thresholds for biopsy decision-making

**DOI:** 10.1007/s00330-025-12272-y

**Published:** 2026-01-04

**Authors:** Selahattin Durmaz, Shun-Chin Jim Wu, Kang-Lung Lee, Amreen Shakur, Iztok Caglic, Tristan Barrett

**Affiliations:** 1 Department of Radiology, Gaziosmanpasa Training and Research Hospital, Istanbul, Turkey; 2 Department of Psychiatry, University of Cambridge, Cambridge, United Kingdom; 3 Department of Radiology, University of Cambridge, Cambridge, United Kingdom; 4 Department of Radiology, Taipei Veterans General Hospital, Taipei City, Taiwan; 5 School of Medicine, College of Medicine, National Yang Ming Chiao Tung University, Taipei City, Taiwan; 6 Department of Radiology, Cambridge University Hospitals NHS Foundation Trust Addenbrooke’s Hospital, Cambridge, United Kingdom; 7 Department of Radiology, University College London, London, United Kingdom

**Keywords:** Magnetic resonance imaging, Prostate cancer, Prostate-specific antigen density, Prostate volume, PI-RADS

## Abstract

**Objectives:**

To evaluate the effect of prostate volume on the risk of clinically significant prostate cancer (csPCa) across a range of PSA-density (PSAd) values, and to explore the relationship between PI-RADS category and PSAd in predicting csPCa.

**Materials and methods:**

We retrospectively analyzed 2190 patients undergoing mpMRI for suspected PCa. Patients were classified as csPCa and clinically insignificant (negative and insignificant PCa). Logistic regression was performed to assess the csPCa risk at different PSAd cut-offs across different prostate volume subgroups (< 40, 40–60, 60–80, > 80 mL) and PI-RADS categories. The effect of prostate volume on PSAd performance was evaluated using ROC analysis. To assess robustness, we performed an 80:20 split-sample internal validation.

**Results:**

747/2190 (34.1%) patients had PCa, including 571 (26.1%) with csPCa. Regardless of PSAd, csPCa risk exceeded 30% for PI-RADS 4 and 50% for PI-RADS 5. At a 10% csPCa risk threshold, the optimal PSAd cut-offs were 0.20 ng/mL² for PI-RADS 1–2 and 0.12 ng/mL² for PI-RADS 3. Logistic regression showed a significant inverse correlation between prostate volume and csPCa probability. Notably, 79% of csPCa patients with prostate volume ≤ 40 mL had a PSAd ≥ 0.15 ng/mL², compared to only 22.4% with volumes ≥ 60 mL. PSAd performed significantly worse for larger glands (≥ 60 mL), with AUCs of 0.70 versus 0.84 (≤ 40 mL) and 0.82 (40–60 mL), both *p* < 0.001.

**Conclusion:**

The optimal PSAd cut-offs for guiding biopsy decisions were 0.20 ng/mL² for PI-RADS 1–2 and 0.12 ng/mL² for PI-RADS 3. When using PSAd to guide biopsy decision for PI-RADS 1–3 patients with large prostates (> 60 mL), caution is warranted, as PSAd becomes significantly less accurate.

**Key Points:**

***Question**** The optimal PSA-density thresholds for biopsy decisions in PI-RADS 1–3 patients remain uncertain, and data on the impact of prostate volume on its performance are limited*.

***Findings**** Optimal PSA-density thresholds were 0.20 ng/mL² for PI-RADS 1–2 and 0.12 ng/mL² for PI-RADS 3. Diagnostic performance of PSA-density decreased significantly in men with larger glands (≥ 60 mL)*.

***Clinical relevance**** PSA-density cut-offs of 0.20 (PI-RADS 1–2) and 0.12 ng/mL² (PI-RADS 3) can guide biopsy decisions. In PI-RADS 1–3 patients with large prostate (≥ 60 mL), PSA-density becomes significantly less predictive, and low values may not reliably exclude csPCa*.

**Graphical Abstract:**

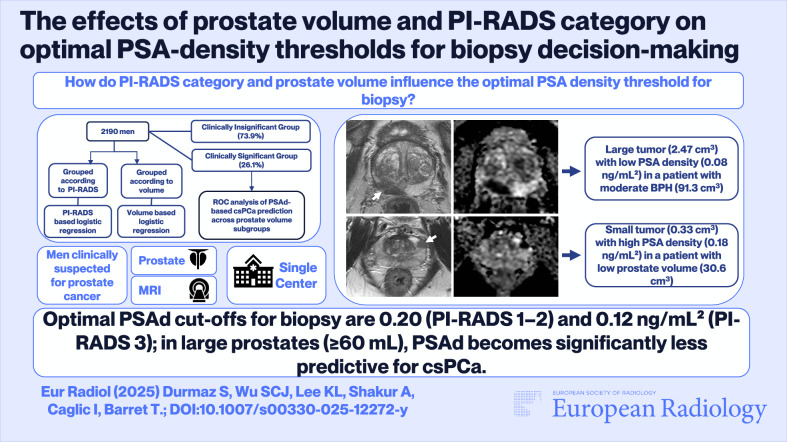

## Introduction

Multiparametric MRI (mpMRI) is now established for the diagnostic work-up of prostate cancer (PCa) [1], having been shown to reduce the number of unnecessary biopsies, improve the detection of clinically significant prostate cancer (csPCa), and limit the overdiagnosis and overtreatment of clinically insignificant disease [2, 3].

The success of mpMRI is primarily driven by its high negative predictive value for excluding csPCa [4], with 28–36% of men with low-likelihood MRI findings (PI-RADS ≤ 2) being able to avoid immediate biopsy [2, 3, 5]. However, due to the low positive predictive value (PPV) of mpMRI, a significant proportion of patients with positive MRI findings at a threshold of PI-RADS score ≥ 3 still undergo unnecessary biopsies [6–8]. According to multicentre studies [2, 6] and systematic reviews [7, 8], the detection rate of csPCa (ISUP Grade Group (GG) ≥ 2) in patients with PI-RADS ≥ 3 lesions ranges from 35% to 45%. In other words, approximately 55–65% of patients with a positive mpMRI either have negative biopsies or are diagnosed with clinically insignificant PCa (cisPCa). The relatively low PPV of mpMRI is particularly driven by the low csPCa detection rate in PI-RADS 3 lesions, which ranges from 12% to 18.5% [2, 6–8].

Integrating clinical parameters with MRI findings may help to avoid unnecessary biopsies, with PSA-density (PSAd) emerging as a useful biomarker to augment biopsy decision-making for patients with PI-RADS scores of 1–3. However, there remains no consensus on the optimal PSAd cutoff for recommending biopsy [9]. Several studies have proposed a PSAd threshold of 0.15 ng/mL² to guide biopsy decision in patients with negative (PI-RADS 1–2) [10–12] or equivocal (PI-RADS 3) MRI findings [10, 11, 13]. This PSAd threshold is also cited in the current European Association of Urology [14] and UK National Institute of Clinical Excellence (NICE) [15] guidelines as indicative of a higher risk of csPCa. However, prior studies have either used a single PSAd cutoff or evaluated a limited set of predefined PSAd cutoff values rather than assessing the entire range of PSAd to justify the use of 0.15 ng/mL² as the optimal threshold [10–13]. Moreover, this threshold was established in the pre-MRI era to identify individuals at high risk for csPCa, and both its origin and underlying rationale remain unclear [16]. Therefore, the optimal PSAd threshold for biopsy decision-making for each PI-RADS category should be further investigated through quantitative analyses assessing the entire range of PSAd values and across PI-RADS categories [17, 18]. In addition, both PSA levels and PSAd will be affected by the volume of benign prostatic hypertrophy (BPH), yet only a limited number of studies have assessed the effects of prostate volume on the predictive performance of PSAd for csPCa [19–21].

In this study, we evaluated the risk of csPCa across a range of PSAd values within each PI-RADS category, aiming to identify the most appropriate cutoff to safely defer immediate biopsy for each PI-RADS group, based on a clinically accepted 10% risk threshold for csPCa. Additionally, we assessed the impact of prostate volume on the predictive performance of PSAd for csPCa.

## Materials and methods

### Study population

This single-center retrospective study was approved by the Local Ethics Committee (HRA and Health and Care Research Wales, IRAS #313163), with the requirement for written informed consent waived.

Between January 2018 and April 2024, a total of 3727 men suspected of having csPCa underwent mpMRI. The following exclusion criteria were applied: PSA > 30 ng/mL (*n* = 105); clinical or pathological diagnosis of prostatitis (*n* = 341), prior intervention or medication for BPH (*n* = 82), previous prostate biopsy (*n* = 18), prior diagnosis of PCa (*n* = 7), and poor MRI quality, defined as a Prostate Imaging Quality (PI-QUAL) score of ≤ 2 (*n* = 37), using version 1 [22], which was the version available at the time of analysis. The remaining patients were categorized as mpMRI-positive (PI-RADS ≥ 3) and mpMRI-negative (PI-RADS 1–2).

Additional exclusion criteria were applied for low-risk PI-RADS 1–2 patients who had no biopsy and less than 12 months of follow-up (*n* = 450) and for mpMRI-positive patients: non-biopsied PI-RADS 4/5 patients (*n* = 88), more than one target lesion (*n* = 353), diffuse involvement on mpMRI (*n* = 4), and metastatic findings (*n* = 43). Non-biopsied, low-risk PI-RADS 3 patients with less than 12 months of follow-up were also excluded (*n* = 9).

**Fig. 1:**
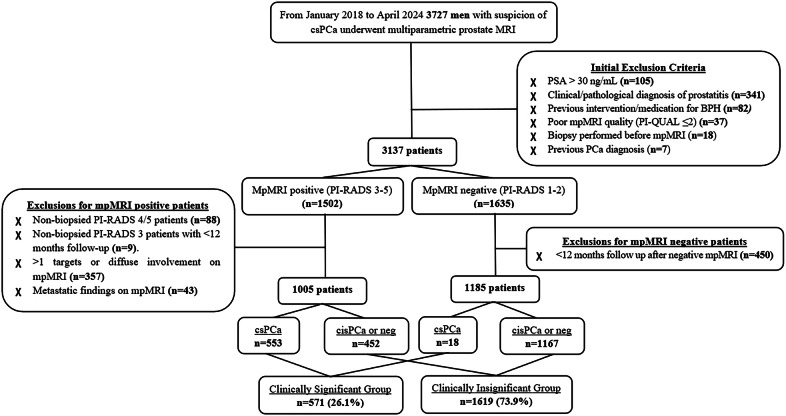
Study flowchart. BPH, benign prostatic hyperplasia; csPCa, clinically significant prostate cancer; cisPCa, clinically insignificant prostate cancer; neg, negative

The final study cohort included 2190 patients. Those diagnosed with csPCa, defined as ISUP GG ≥ 2, were classified into the clinically significant group, while patients with negative biopsy results or clinically insignificant PCa were categorized into the clinically insignificant group. The reference standard was based on pathology results from MRI-guided fusion biopsy and systematic biopsy. Additionally, low-risk patients (PI-RADS 1–3) who were not biopsied but completed at least 12 months of follow-up without evidence of PCa progression were also included in the insignificant group. A flowchart illustrating the stepwise selection process is presented in Fig. 1.

### Magnetic resonance imaging acquisition

All mpMRI examinations were carried out using one of the following five MR systems: 1.5-T Discovery MR450, 1.5-T Optima MR450w, 1.5-T SIGNA Artist, 3.0-T Discovery MR750, and 3.0-T SIGNA Premier (GE Healthcare), all equipped with a 16–32 channel surface phased array coil. The imaging acquisition protocol has been previously detailed by Karanasios et al [23]. In summary, each system followed a standardized PI-RADS compliant protocol that included: (1) axial Fast Spin Echo T1-weighted imaging (WI) of the pelvis, (2) small field-of-view (FOV), high-resolution T2-weighted Fast Recovery Fast Spin Echo imaging of the prostate in axial, coronal, and sagittal planes, (3) axial high b-value (b1400 at 1.5 T, b2000 at 3 T) diffusion-weighted imaging (DWI) aligned with axial T2-WI, with automatically generated apparent diffusion coefficient (ADC) maps, and (4) axial dynamic contrast-enhanced images, also aligned with axial T2-WI, performed after a bolus injection of Gadobutrol (Gadovist, Bayer HealthCare) at a rate of 3 mL/s (dose: 0.1 mmol/kg) via a power injector. The detailed MRI acquisition protocol is provided in Table S1.

### Image analysis

Multiparametric prostate MRIs were interpreted as part of the standard clinical workflow following PI-RADS v2 [24] (2015–2019) and PI-RADS v2.1 [25] (2019-present) by subspecialist uroradiologists with 7–14 years of experience in prostate MRI, all of whom were considered experts [26, 27]. Prostate volume and lesion analysis were performed using DynaCAD (Invivo, Philips Healthcare).

### Biopsy

Biopsies were performed via a transrectal or transperineal approach using MRI/ultrasound fusion. All procedures were performed by experienced urologists and included targeted and systematic sampling. Transrectal biopsies were conducted in an outpatient setting using the UroNav fusion platform (InVivo Corp.). Transperineal biopsies were performed using the Biopsee fusion system (Medcom). In cases of negative MRI, biopsy decisions were made on an individual basis following a clinical risk assessment. Patients who did not undergo biopsy were monitored through a safety-net follow-up strategy, with a least one PSA test performed at 6–12 months.

### Statistical analyses

R version 4.2.3 (R Foundation for Statistical Computing) was used for statistical analysis. Continuous variables were reported as medians with interquartile ranges (IQR), while categorical variables were presented as counts and percentages. The Mann–Whitney U test was used to compare continuous variables, and the Chi-Square test was employed to assess differences in categorical variables between the significant and insignificant groups.

The entire cohort was grouped according to PI-RADS scores and predefined prostate volume categories of < 40 mL, 40–60 mL, 60–80 mL, and > 80 mL. These volume categories were determined a priori based on the expected distribution in a symptomatically referred patient population and therefore reflective of clinically meaningful thresholds. These cut-offs are consistent with prior studies assessing PSAd in relation to prostate size [20, 28, 29]. Separate univariable logistic regression analyses were performed based on PI-RADS scores and prostate volume to evaluate the risk of csPCa at different PSAd cutoff values across these subgroups. Given that few urologists would prefer sampling more than 10 men to diagnose a single case of csPCa [30], and that multiple MRI-based risk models demonstrate net clinical benefit at csPCa risk thresholds of ≥ 10% for biopsy [31], we selected a 10% csPCa probability threshold to define the optimal PSAd cutoff for each PI-RADS group. In a subgroup analysis of patients diagnosed with csPCa, individuals were stratified by prostate volume (≤ 40 mL, 40–60 mL, and ≥ 60 mL) and by PSAd levels (< 0.15 ng/mL² vs ≥ 0.15 ng/mL²). The proportion of men with PSAd < 0.15 ng/mL² and ≥ 0.15 ng/mL² was calculated for each volume group and compared by using the Chi-Square test.

All univariable logistic regression analyses were conducted using the stats package (version 4.2.3), and model assumptions were evaluated using the performance package (version 0.12.3). To compare the predicted probability of csPCa at a specific PSAd threshold (0.15 ng/mL²) between PI-RADS categories and preselected prostate volume subgroups, we used bootstrapped differences (5000 iterations), with statistical significance determined by whether the 95% confidence interval excluded 0 and by testing against the null hypothesis of no difference (alpha = 0.05).

Receiver operating characteristic (ROC) curves were generated for models using PSAd to predict csPCa across prostate volume subgroups (≤ 40 mL, 40–60 mL, ≥ 60 mL). We calculated the area under the ROC curve (AUC-ROC) with 95% confidence intervals, Youden’s index, sensitivity, and specificity at specific PSAd thresholds (0.12, 0.15, and 0.20 ng/mL^2^) using the pROC package (version 1.18.5). Comparisons of AUC-ROC values between prostate volume subgroups (≤ 40 mL, 40–60 mL, ≥ 60 mL) were performed using both the DeLong test [32] and bootstrapping (5000 iterations). Differences in sensitivity and specificity at defined PSAd thresholds between groups were similarly evaluated using bootstrapped comparisons (5000 iterations), with significance assessed as above. To internally validate our findings and assess the robustness of the results, we performed a split-sample internal validation (80:20) using the *caTools* package (version 1.18.3) in R as a sensitivity analysis.

## Results

The final study cohort consisted of 2190 patients, with a median age of 66 years (IQR: 61–72 years), a median PSA level of 5.6 ng/mL (IQR: 4–8 ng/mL), a median prostate volume of 56 mL (IQR: 39–80 mL), and a median PSAd of 0.10 ng/mL² (IQR: 0.07–0.14 ng/mL²). A total of 1073 patients underwent biopsy. The overall PCa rate was 34.1% (*n* = 747/2190), with a 26.1% (*n* = 571/2190) prevalence of csPCa. The remaining 1117 non-biopsied low-risk patients (PI-RADS 1–2: 94.8%, *n* = 1059; PI-RADS 3: 5.2%, *n* = 58) were followed up for a median of 33.8 months (IQR: 26.2–49.5 months) without evidence of progression and were therefore considered negative for csPCa. The median PSAd values of the significant and insignificant groups were 0.18 ng/mL² (IQR: 0.12–0.27 ng/mL²) and 0.08 ng/mL² (IQR: 0.06–0.11 ng/mL²), respectively (*p* < 0.001). The median values for age, PSA, PSAd, and prostate volume for both groups are presented in Table 1. The csPCa detection rates for each PI-RADS category were as follows: 1.5% (*n* = 18/1185) for PI-RADS 1–2, 10.7% (*n* = 23/215) for PI-RADS 3, 50.1% (*n* = 190/379) for PI-RADS 4 and 82.7% (*n* = 340/411) for PI-RADS 5.

**Table 1:** Baseline characteristics of patients in the clinically significant and clinically insignificant groups

Characteristics	Clinically significant group (*n* = 571; 26.1%)	Clinically insignificant group (*n* = 1619; 73.9%)	*p*-value
Age	68 (64–74)	65 (60–70)	< 0.001
PSA (ng/mL)	7.1 (5.1–10.9)	5.2 (3.9–7.2)	< 0.001
Prostate volume (mL)	40 (30–55)	62.4 (44.1–87.8)	< 0.001
PSAd (ng/mL^2^)	0.18 (0.12–0.27)	0.08 (0.06–0.11)	< 0.001
PI-RADS score, *n* (%)			< 0.001
1, 2	18 (3.2)	1167 (72.1)	
3	23 (4)	192 (11.8)	
4	190 (33.3)	189 (11.7)	
5	340 (59.5)	71 (4.4)	
Gleason grade group, *n* (%)			< 0.001
Negative	0 (0)	1443 (70.6)	
1	0 (0)	176 (29.4)	
2	324 (56.7)	0 (0)	
3	109 (19.1)	0 (0)	
4	50 (8.8)	0 (0)	
5	88 (15.4)	0 (0)	

Estimates were given as median (quartile 1, quartile 3) or frequency (percentage)

*PSA* prostate-specific antigen, *PSAd* prostate-specific antigen density

* *p*-values were calculated using the Mann–Whitney U test for continuous and the Chi-Square test for categorical variables

### Logistic regression analysis based on PI-RADS scores

**Fig. 2:**
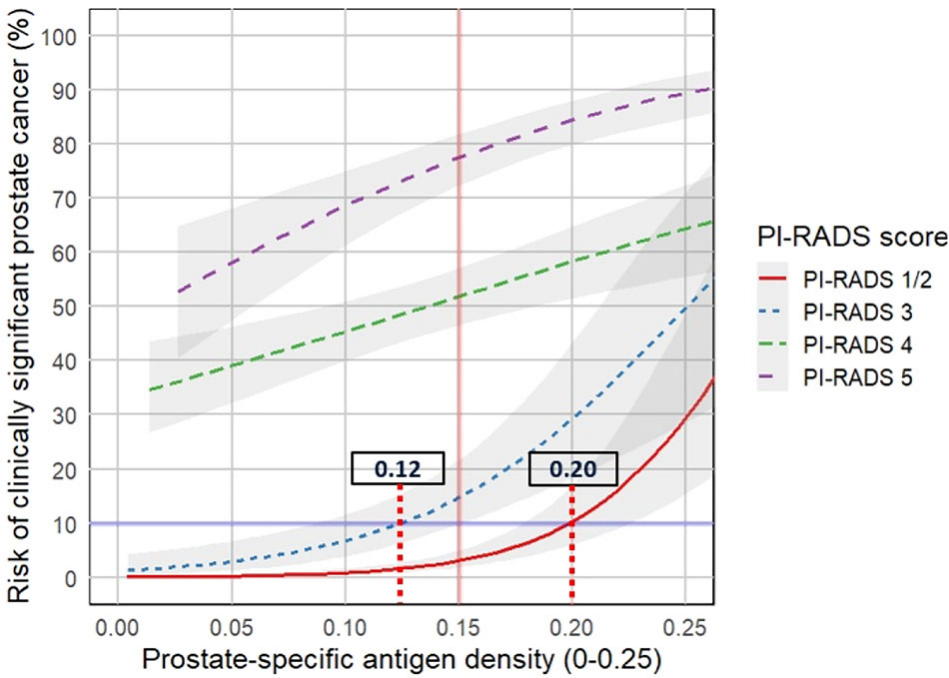
Probability of clinically significant prostate cancer across a range of PSA-density (PSAd) values stratified by PI-RADS category. Dashed red vertical lines indicate the optimal PSAd cutoff values of 0.20 ng/mL² and 0.12 ng/mL² for biopsy decision-making in PI-RADS 1–2 and PI-RADS 3 patients, respectively, corresponding to a 10% csPCa probability threshold (solid blue horizontal line). The solid red vertical line indicates PSAd = 0.15 ng/mL², a commonly used clinical cutoff. csPCa, clinically significant prostate cancer

Single-variable LR analysis indicated that, regardless of PSAd, the likelihood of csPCa exceeded 30% for PI-RADS 4 and 50% for PI-RADS 5 patients (Fig. 2). At a PSAd cutoff of 0.15 ng/mL², the probability of csPCa for each PI-RADS category was as follows: 3% for PI-RADS 1–2, 14.8% for PI-RADS 3, 52% for PI-RADS 4, and 77.4% for PI-RADS 5. Based on a 10% risk threshold for csPCa, the optimal PSAd cut-offs for PI-RADS 1–2 and PI-RADS 3 scores were identified as 0.20 ng/mL² and 0.12 ng/mL², respectively (Fig. 2). Among patients with PI-RADS 3 lesions, the csPCa detection rate was 2.8% in those with a PSAd < 0.12 ng/mL², compared to 26.4% in those with a PSAd ≥ 0.12 ng/mL². Similarly, in patients with PI-RADS 1–2 MRI findings, the csPCa detection rate was 1.2% for the group with PSAd < 0.20 ng/mL² and 17.9% for the group with PSAd ≥ 0.20 ng/mL² (Fig. S1).

### Logistic regression analysis based on prostate volumes

**Fig. 3:**
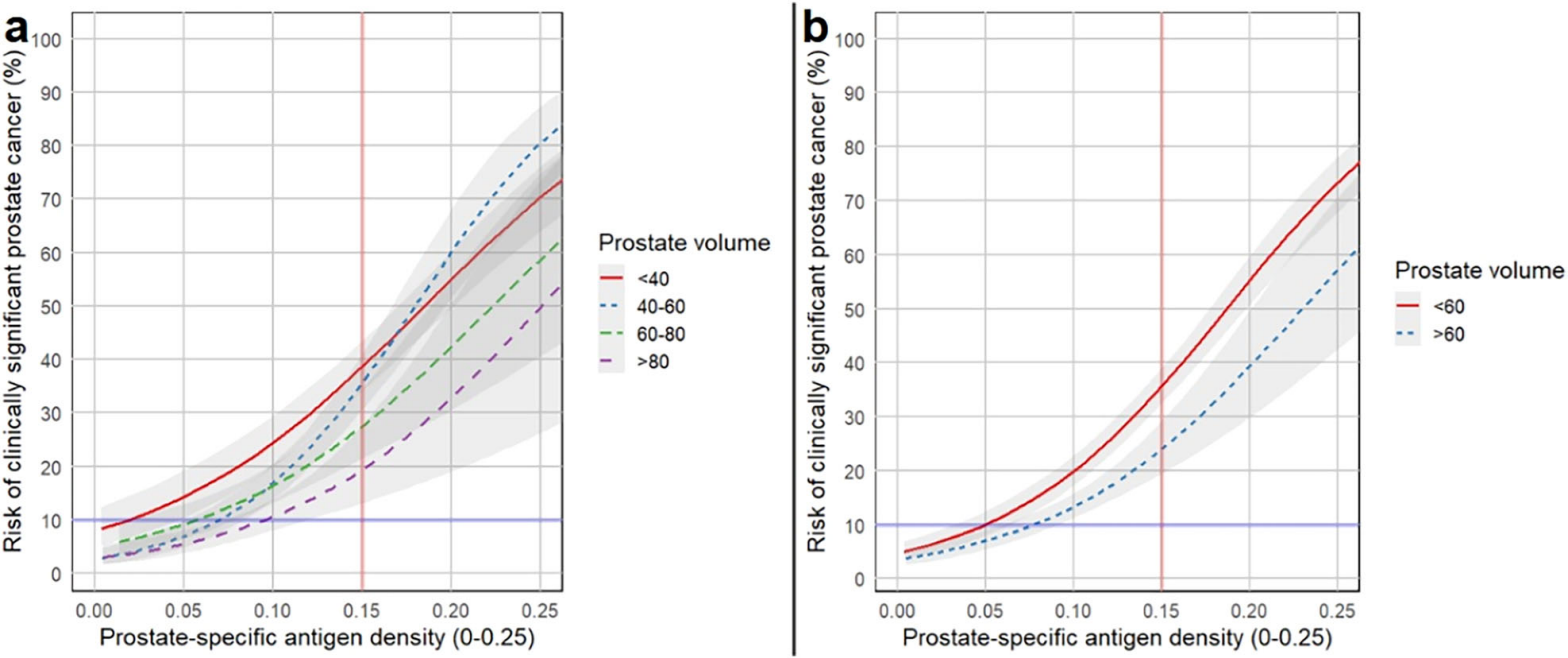
Probability of clinically significant prostate cancer across the full range of PSA-density (PSAd) values, stratified by prostate volume subcategories. **a** < 40 mL, 40–60 mL, 60–80 mL, and > 80 mL; **b** < 60 mL vs > 60 mL. The solid red vertical line indicates PSAd = 0.15 ng/mL², a commonly used clinical cutoff. The solid blue horizontal line marks the 10% csPCa risk threshold

Two separate LR analyses were performed based on prostate volume subgroups. In the primary analysis, patients were categorized into four prostate volume groups: < 40 mL, 40–60 mL, 60–80 mL, and > 80 mL. This analysis revealed an inverse correlation between prostate volume and the probability of csPCa at a PSAd threshold of 0.15 ng/mL²: 38.6% csPCa probability for volume < 40 mL, 35.6% for 40–60 mL, 27.5% for 60–80 mL, and 19.3% for > 80 mL (Fig. 3a). The clearest separation occurred at a prostate volume threshold of 60 mL, (Fig. 3b), with 35.6% csPCa probability for patients with prostate volumes < 60 mL and 24% for those with volumes > 60 mL.

**Fig. 4:**
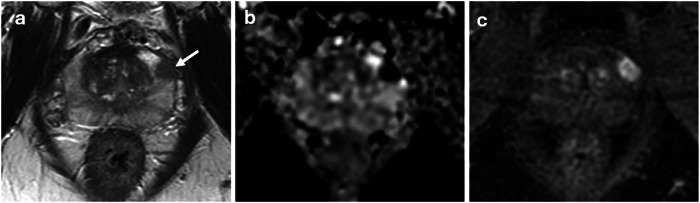
Small tumor with high PSA density in a patient with a low-volume prostate. 75-year-old patient with PSA 5.61 ng/mL. 10 × 7 × 9 mm (volume 0.33 cm^3^) PI-RADS 4 lesion at the left anterior mid gland base PZ on T2WI (arrow in **a**). Marked restricted diffusion on ADC (**b**) and early enhancement on DCE (**c**). Gland volume 30.6 cm^3^, high PSA-density of 0.18 ng/mL^2^. Biopsy shows Gleason 4 + 3 = 7 (pattern 4 = 70%). Patient treated with external beam radiotherapy. ADC, absolute diffusion coefficient; DCE, dynamic contrast-enhanced; PZ, peripheral zone

**Fig. 5:**
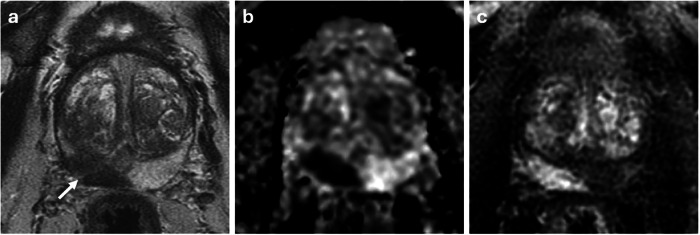
Large tumor with low PSA density in a patient with moderate BPH. 75-year-old patient with PSA 7.48 ng/mL. 20 × 11 × 21 mm (volume 2.47 cm^3^) PI-RADS 5 lesion at the right base PZ on T2WI (arrow in **a**). Marked restricted diffusion on ADC (**b**) and early enhancement on DCE (**c**). Gland volume 91.3 cm^3^, low PSA-density of 0.08 ng/mL^2^. Biopsy shows Gleason 4 + 3 = 7 (pattern 4 = 60%). Patient treated with external beam radiotherapy. ADC, absolute diffusion coefficient; DCE, dynamic contrast-enhanced; PZ, peripheral zone

**Fig. 6:**
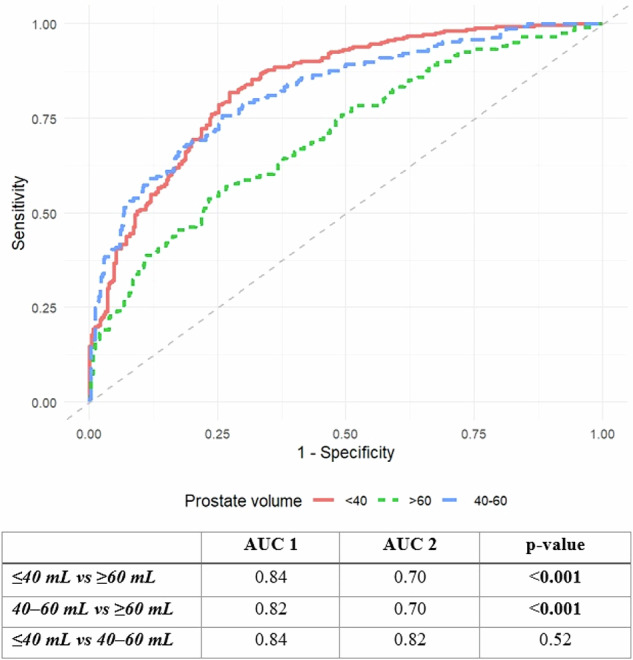
Receiver operating characteristic (ROC) analysis demonstrating the predictive performance of PSA-density for detecting clinically significant prostate cancer in prostate volume subgroups (small (≤ 40 mL), medium (40–60 mL), and large (≥ 60 mL)). AUC values and pairwise comparisons are summarized in the table below

In a further subgroup analysis, we also assessed the distribution of csPCa patients across various prostate volume subgroups (≤ 40 mL, 40–60 mL, ≥ 60 mL) based on PSAd (< 0.15, ≥ 0.15 ng/mL²). In csPCa patients with small prostate volume (≤ 40 mL), 21% (61/291) had a PSAd < 0.15 ng/mL², whereas 79% (230/291) had a PSAd of ≥ 0.15 ng/mL². By contrast, only 22.4% (28/125) of patients with csPCa and gland volume ≥ 60 mL had PSAd ≥ 0.15 ng/mL² (Fig. S2). Representative csPCa cases with small and large prostate volumes illustrating these contrasting PSAd patterns are presented in Figs. 4 and 5. Moreover, ROC analysis confirmed that the predictive performance of PSAd for csPCa was significantly lower in patients with larger glands (≥ 60 mL) showing AUC at 0.70, versus AUCs of 0.84 (≤ 40 mL) and 0.82 (40–60 mL), (*p* < 0.001 for both comparisons); Fig. 6. At PSAd thresholds of 0.12, 0.15, and 0.20 ng/mL², the highest sensitivity and lowest specificity were observed in the small-volume group (≤ 40 mL), whereas the lowest sensitivity and highest specificity were seen in the large-volume group (≥ 60 mL) (Table 2). These differences were statistically significant (Table S2).

**Table 2:** Sensitivity and specificity of PSA-density thresholds (0.12, 0.15, and 0.20 ng/mL²) for detecting clinically significant prostate cancer across prostate volume subgroups (small (≤ 40 mL), medium (40–60 mL), and large (≥ 60 mL))

Prostate volume group	PSAd threshold (ng/mL^2^)	Sensitivity (%)	Specificity (%)
≤ 40 mL	0.12	93	53
	0.15	80	73
	0.20	59	85
40–60 mL	0.12	71	77
	0.15	56	90
	0.20	36	97
≥ 60 mL	0.12	39	89
	0.15	23	95
	0.20	15	99

*PSAd* prostate-specific antigen density

### Internal validation

The split-sample internal validation (80:20) demonstrated results consistent with the full-dataset analysis, confirming the robustness of the findings. However, the reduced sample sizes in certain subgroups (e.g., prostate volume > 60 cc) limited statistical significance and resulted in wider confidence intervals, which should be interpreted with caution (Figs. S3–S5).

## Discussion

In the present study, we observed a high baseline risk of csPCa in patients with PI-RADS 4 (> 30%) and PI-RADS 5 (> 50%) lesions, regardless of PSAd, which always warrant biopsy. However, using a csPCa probability threshold of 10%, the optimal PSAd cutoff values of 0.20 ng/mL² could be applied to augment the decision to biopsy in patients with PI-RADS 1–2 scores and 0.12 ng/mL² for PI-RADS 3 lesions. Additionally, we demonstrated that prostate volume significantly affects the predictive performance of PSAd for csPCa; 79% csPCa patients with prostate volumes ≤ 40 mL had a PSAd ≥ 0.15 ng/mL², compared to only 22.4% of those with prostate volumes ≥ 60 mL, suggesting that the conventional PSAd cutoff of 0.15 ng/mL² should be used with caution for patients with larger glands.

PSAd has been shown to be a significant independent predictor of csPCa at biopsy [11] and can be combined with pre-biopsy MRI findings in patients with negative or equivocal MRI results to aid biopsy decision-making [9]. Consistent with our findings, previous studies have shown that PSAd alone does not provide sufficient reassurance to safely defer biopsy in patients with PI-RADS 4–5 lesions [17, 18, 33]. In contrast, it has been recognized as a useful tool for risk stratification in patients with PI-RADS scores 1–3 by reducing unnecessary biopsies and the overdiagnosis of cisPCa [33]. PSAd of 0.15 ng/mL² as a predefined cutoff has been demonstrated to be beneficial in PI-RADS 1–2 patients [10–12]. However, Pellegrino et al [16] concluded that a PSAd threshold of ≥ 0.20 ng/mL² is more appropriate when using 10% as an optimal csPCa probability threshold, while 0.15 ng/mL² may be justified only when MRI accuracy is substantially limited. Likewise, numerous studies [10–13, 17] have also proposed PSAd of 0.15 ng/mL² as an optimal threshold for guiding biopsy decisions in PI-RADS 3 patients. However, the majority of these studies either employed a single PSAd cutoff or assessed only a limited number of preselected thresholds, rather than evaluating the entire range of PSAd values. Vanderick et al [17] demonstrated that applying a PSAd cutoff of 0.15 ng/mL² for PI-RADS 3 lesions could avoid biopsy in 42% of cases, with a 6% miss rate for csPCa, whereas a lower threshold of 0.12 ng/mL² avoided 26% of biopsies without missing any csPCa. Similarly, Pellegrino et al [18] recommended a threshold of 0.10 ng/mL² for safely deferring biopsy in PI-RADS 3 lesions, based on a 10% csPCa risk threshold. Schoots et al [34] proposed PSAd thresholds of 0.20 ng/mL² and 0.10 ng/mL² as low-risk criteria to safely defer immediate biopsy in patients with PI-RADS 1–2 scores and PI-RADS 3 lesions, respectively. This approach was validated in a subsequent study [35], with the highest net benefit balancing biopsy avoidance and csPCa detection achieved when biopsying PI-RADS 3 lesions at PSAd ≥ 0.10–0.15 ng/mL², and for PI-RADS 1–2 lesions at PSAd > 0.20 ng/mL. Our findings support these risk-adapted strategies but suggest a slightly higher PSAd threshold (≥ 0.12 ng/mL²) for PI-RADS 3 lesions. This may be attributed to the lower csPCa prevalence among PI-RADS 3 patients in our cohort (10.5% vs 16–17.5% in prior studies), as well as our use of a range of PSAd values rather than predefined cut-offs.

We also evaluated the predictive performance of PSAd cut-offs of 0.12, 0.15 and 0.20 ng/mL² across prostate volume subgroups and observed the highest sensitivity and lowest specificity in the small-volume group (≤ 40 mL, AUC: 0.84) and lowest sensitivity and highest specificity in the large-volume group (≥ 60 mL, AUC: 0.70). Limited studies have assessed the relationship between prostate volume and the predictive performance of PSAd for csPCa, with findings consistent with our work [19–21]. Omri et al [21] reported the highest proportion of men with csPCa and PSAd ≥ 0.15 ng/mL² in the small-volume group (≤ 50 mL), while the lowest was seen in the large-volume group (≥ 75 mL). Two recently published studies [19, 20] concluded that the diagnostic performance of PSAd is significantly influenced by prostate volume, with the lowest accuracy observed in large-volume groups (≥ 70 mL [19] and ≥ 50 mL [20]). At the PSAd threshold of 0.15 ng/mL², all three studies reported the highest sensitivity and lowest specificity in small-volume groups, and the lowest sensitivity and highest specificity in large-volume groups [19–21]. Furthermore, Robinson et al [20] also evaluated two additional PSAd cut-offs (0.10 and 0.20 ng/mL), observing similar trends. Given the reduced predictive performance of PSAd in men with large prostate volumes, complementary tools may be utilized to improve risk assessment. In men with PI-RADS 1–3 findings, large prostate volume, and low PSAd, risk prediction and biopsy decision-making may be refined using validated MRI-integrated risk calculators, such as Prospective Loyola University Multiparametric MRI (PLUM) [36], and Rotterdam Prostate Cancer Risk Calculator–MRI (RPCRC-MRI) [37, 38]. Furthermore, serum- and urine-based biomarkers, including the Prostate Health Index (PHI), 4Kscore, and SelectMDx, may be incorporated into the diagnostic pathway for this patient group, providing additional risk stratification beyond MRI and clinical parameters [39, 40].

Our study has some limitations, including the retrospective design. This study was conducted at a single, high-volume tertiary care center with experienced radiologists, which may limit the generalizability of our findings. The robustness of our approach was supported by internal validation; however, external validation in independent cohorts is encouraged to confirm the broader applicability of our results. Although PI-RADS-compliant imaging protocols were used, mpMRI examinations were carried out using five different MR systems with two field strengths (1.5 T and 3.0 T). We acknowledge that this heterogeneity in MRI acquisition may have influenced PI-RADS assessment and, consequently, its association with PSAd. The proportion of patients with PI-RADS 3 lesions was relatively low in our cohort (9.8%), but comparable to previously reported, similar clinical settings [41]. The use of a 10% csPCa probability threshold to define optimal PSAd cut-offs may not be appropriate in all clinical settings; however, alternative thresholds can be dynamically derived from the plots presented (Fig. 2). Finally, biopsy results were not available for low-risk patients with PI-RADS 1–3 MRI findings. However, all patients were monitored through a safety-net follow-up strategy, with a median time of 33.8 months. Our study benefits from including a large number of patients and evaluating a range of PSAd values to identify the optimal cut-offs for biopsy decision-making and further explores the effect of prostate volume on the diagnostic performance of PSAd for csPCa. Further studies could focus on prospectively validating our findings across multiple centers and among radiologists with varying levels of experience to confirm their generalizability and clinical utility.

## Conclusion

Our study shows that patients with PI-RADS ≥ 4 lesions should undergo biopsy regardless of PSAd value; however, the optimal PSAd cut-offs for guiding biopsy decisions were 0.20 ng/mL² for PI-RADS 1–2 scores and 0.12 ng/mL² for PI-RADS 3 lesions. Furthermore, when using PSAd to guide biopsy decision for PI-RADS 1–3 patients, caution is warranted in those with large prostates (> 60 mL), as PSAd becomes significantly less predictive and low values may provide false reassurance.

## Supplementary information

ELECTRONIC SUPPLEMENTARY MATERIAL

## Abbreviations

AUC-ROC: Area under the ROC curve
BPH: Benign prostatic hypertrophy
cisPCa: Clinically insignificant PCa
csPCa: Clinically significant prostate cancer
IQR: Interquartile ranges
mpMRI: Multiparametric MRI
NICE: National Institute of Clinical Excellence
PI-QUAL: Prostate imaging quality
PSAd: PSA-density
ROC: Receiver operating characteristic
