# Supplementary material for: The effects of prostate volume and PI-RADS category on optimal PSA-density thresholds for biopsy decision-making
Source: Eur Radiol. 2026 Jan 4;36(6):4503–12. doi: 10.1007/s00330-025-12272-y (PMC13212782; doi:10.1007/s00330-025-12272-y)
Supplement: Supplementary file 1 — ELECTRONIC SUPPLEMENTARY MATERIAL [file 330_2025_12272_MOESM1_ESM.pdf]

The Effects of Prostate Volume and PI-RADS Category on Optimal PSA-density Thresholds for Biopsy Decision-Making

ELECTRONIC SUPPLEMENTARY MATERIAL

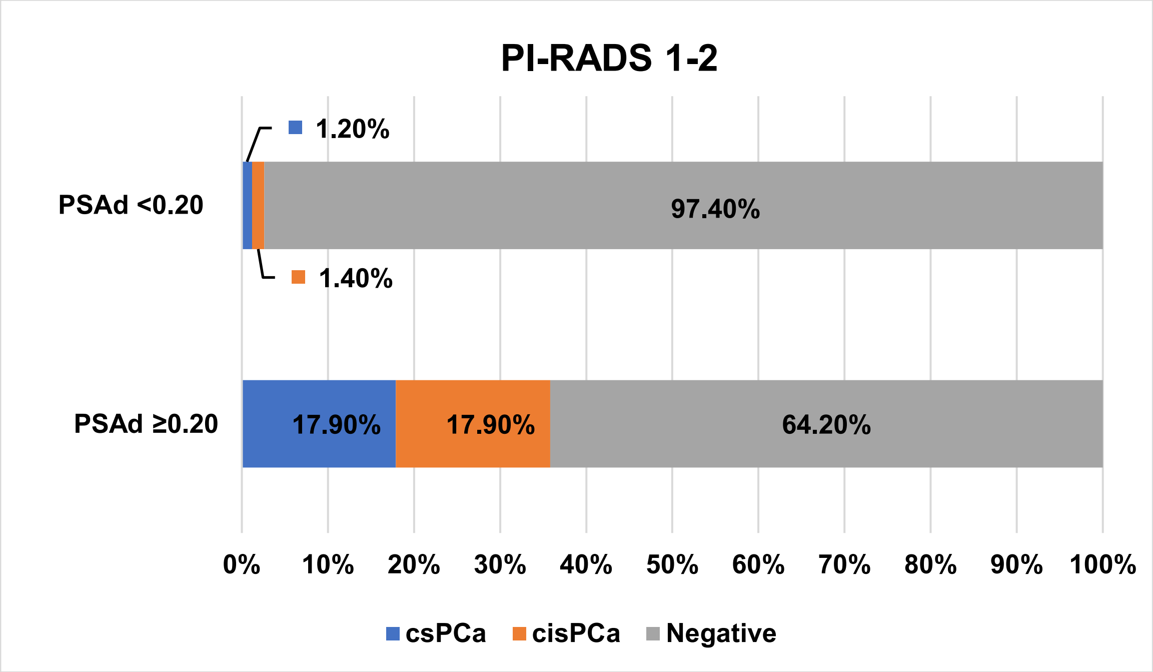

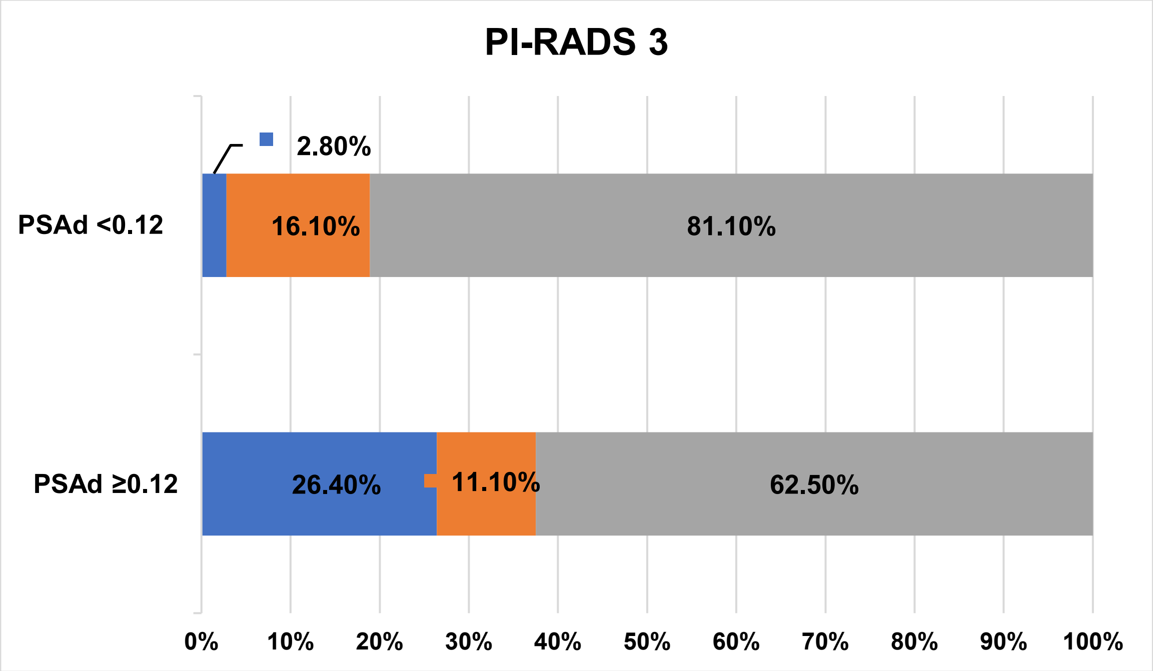

Figure S1: Prevenances of clinically significant prostate cancer (csPCa), clinically insignificant prostate cancer (cisPCa), and negative cases among PI-RADS 1–2 and PI-RADS 3 groups, based on PSA density thresholds of 0.20 ng/mL² and 0.12 ng/mL², respectively.

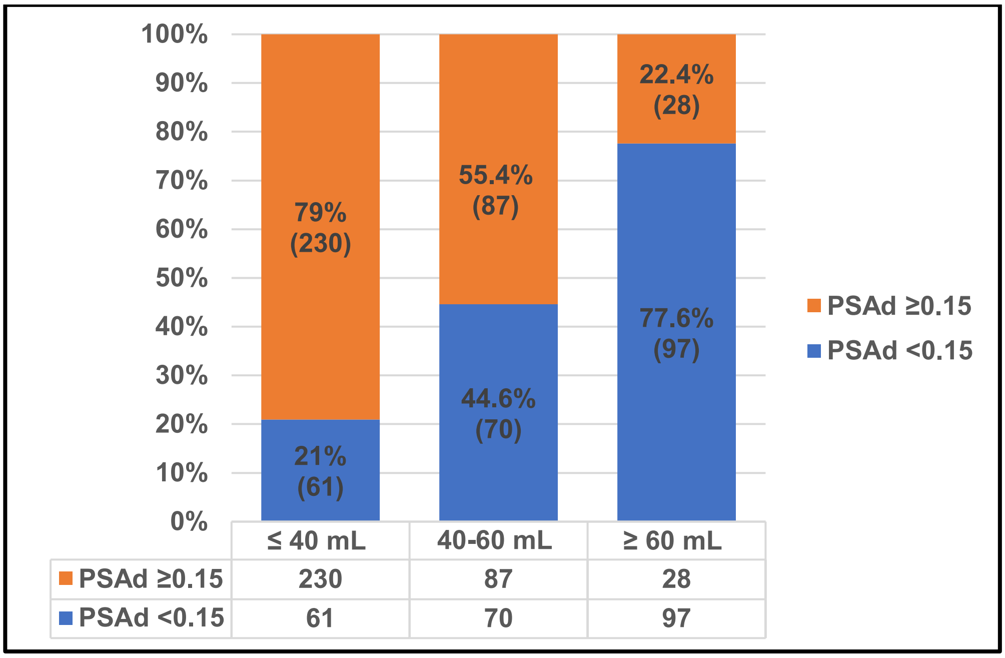

Figure S2: The distribution of patients with clinically significant prostate cancer across various prostate volume subgroups (≤40 mL, 40-60 mL, ≥60 mL) based on PSAd (<0.15, ≥0.15 ng/mL²).

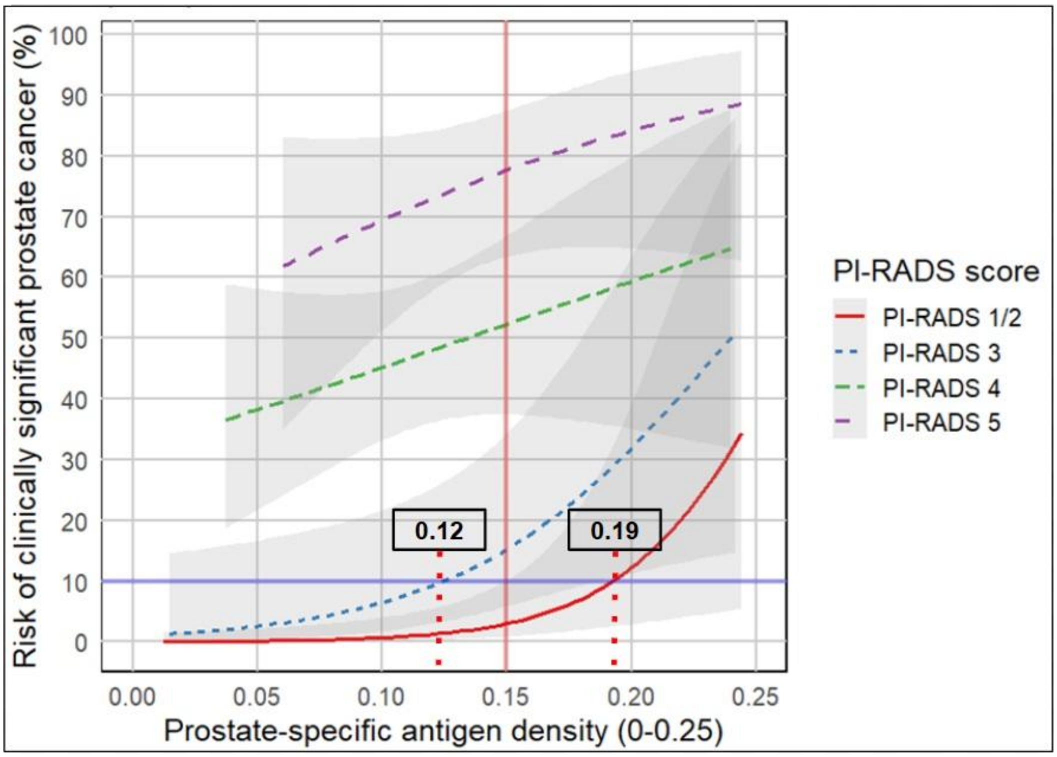

Figure S3: Probability of clinically significant prostate cancer across a range of PSA-density values, stratified by PI-RADS category, derived from the split-sample internal validation (80:20).

Dashed red vertical lines indicate the optimal PSAd cut-off values of 0.19 ng/mL² and 0.12 ng/mL² for biopsy decision-making in PI-RADS 1–2 and PI-RADS 3 patients, respectively, corresponding to a 10% csPCa probability threshold (solid blue horizontal line). The solid red vertical line indicates PSAd = 0.15 ng/mL², a commonly used clinical cut-off.

.

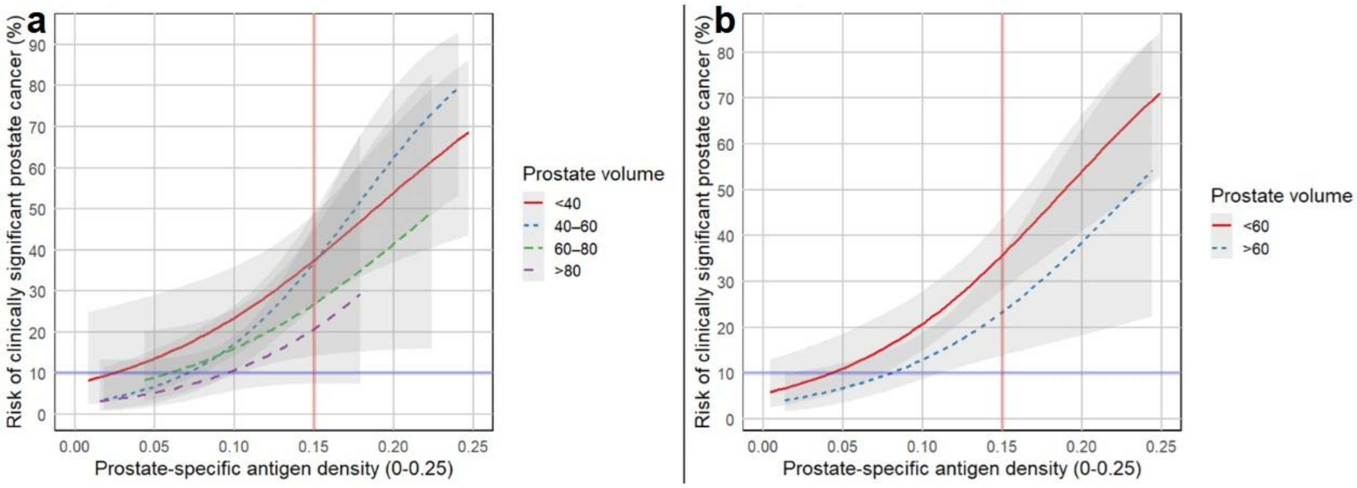

Figure S4: Probability of clinically significant prostate cancer across the full range of PSA-density values, stratified by prostate volume subcategories. **(a)** <40 mL, 40–60 mL, 60–80 mL, and >80 mL; **(b)**. <60 mL vs >60 mL, derived from the split-sample internal validation (80:20).

The solid red vertical line indicates PSAd = 0.15 ng/mL², a commonly used clinical cut-off. The solid blue horizontal line marks the 10% csPCa risk threshold.

.

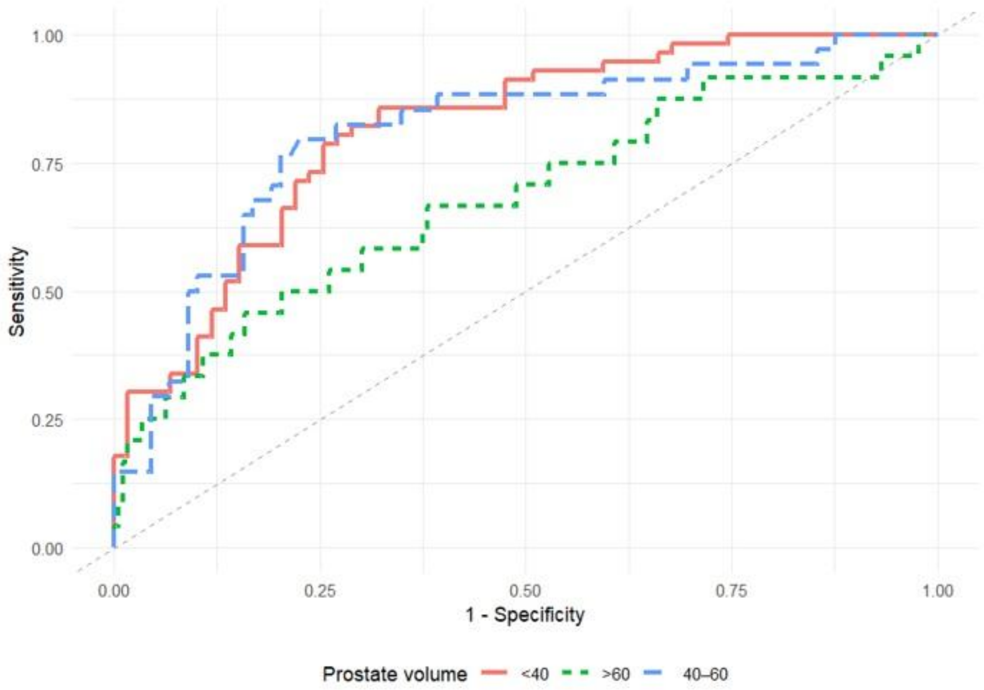

	≤40 mL	40-60 mL	≥ 60 mL
**AUC**	0.82	0.81	0.68

Figure S5: Receiver operating characteristic (ROC) analysis demonstrating the predictive performance of PSA-density for detecting clinically significant prostate cancer in prostate volume subgroups (small (≤40 mL), medium (40–60 mL), and large (≥60 mL)), derived from the split-sample internal validation (80:20). Area-under-the-curve (AUC) values for each subgroup are summarized in the table below.

.

A. Axial T2WI

GE MR System	**Discovery MR450**	**Optima MR450w**	**SIGNA Artist**	**Discovery MR750**	**SIGNA Premier**
Field Strength (T)	1.5	1.5	1.5	3.0	3.0
Receive Coil	32Ch Cardiac	Body 24 AA2<br>Body 30 Small<br>Body 36 AA1	30AA+40PA	32Ch Cardiac	30AA+60PA
Scanning Sequence	SE	SE, PROP	SE, PROP	SE	SE
MR Acquisition Type	2D	2D	2D	2D	2D
Repetition Time (ms)	3418 – 4846	4092 – 7515	2606 – 9128	2500 – 5074	2401 – 3792
Echo Time (ms)	86.0 – 88.4	85.8 – 107.9	79.5 – 87.8	96.8 – 107.1	96.6 – 164.3
Flip Angle (°)	160	160	160	111	111
Number of Averages	2.5	2	1	1.5	1.5
Reconstructed Matrix	512 × 512	512 × 512	512 × 512	512 × 512	512 × 512
Reconstructed Resolution	0.47 × 0.47	0.47 × 0.47	0.35 × 0.35 - 0.41 × 0.41	0.35 × 0.35	0.35 × 0.35
Slice Thickness (mm)	3.0 - 3.5	3.0 - 3.5	3.0	3.0	3.0
Slice Gap (mm)	0.0 - 0.5	0.0 - 0.5	0.0	0.0	0.0
Field of View (cm^2)	24	24	18 - 21	18	18

.

B. Axial DWI

GE MR System	**DISCOVERY MR450**	**Optima MR450w**	**SIGNA Artist**	**DISCOVERY MR750**	**SIGNA Premier**
Field Strength (T)	1.5	1.5	1.5	3.0	3.0
Receive Coil	32Ch Cardiac	Body 24 AA2<br>Body 30 Small<br>Body 36 AA1	30AA+40PA	32Ch Cardiac	30AA+60PA
Scanning Sequence	EP	EP	EP	EP	EP
MR Acquisition Type	2D	2D	2D	2D	2D
Repetition Time (ms)	2634 – 4000	4691 – 7308	4000 – 5963	3321 – 4566	3775 – 4803
Echo Time (ms)	59.2 – 62.0	76.6 – 76.8	72.8 – 87.8	69.2 – 70.3	66.2 – 66.7
Flip Angle (°)	90	90	90	90	90
Acquired Diffusion b Values	I. 50|1400,<br>II. 0|1000	I. 50|1400,<br>II. 50|450|1000	100|550|1000	100|750|1400	100|750|1400
Synthetic Diffusion b Values	I. 2000|2500,<br>II. 1400|2000|2500	I. 2000|2500,<br>II. 1400|2000|2500	1400|2000	2000|2500	2000|2500
Number of Averages per b Value	I. 1|16,<br>II. 1|10	I. 2|16,<br>II. 1|5|12	4|8|12	2|6|6	2|6|6
Reconstructed Matrix	256 × 256	256 × 256	256 × 256	256 × 256	256 × 256
Reconstructed Resolution	0.94 × 0.94	0.94 × 0.94	1.09 x 1.09	1.09 x 1.09	1.09 x 1.09
Slice Thickness (mm)	4.0	4.0	3.0	3.0	3.0
Slice Gap (mm)	0.0	0.0	0.0	0.0	0.0
Field of View (cm^2)	24	24	28	28	28

.

C. Axial DCE

GE MR System	**DISCOVERY MR450**	**Optima MR450w**	**SIGNA Artist**	**DISCOVERY MR750**	**SIGNA Premier**
Field Strength (T)	1.5	1.5	1.5	3.0	3.0
Receive Coil	32Ch Cardiac	Body 24 AA2<br>Body 30 Small<br>Body 36 AA1	30AA+40PA	32Ch Cardiac	30AA+60PA
Scanning Sequence	GR	GR	GR	GR	GR
MR Acquisition Type	3D	3D	3D	3D	3D
Repetition Time (ms)	6.20 – 6.24	6.20 – 6.28	6.16 – 6.85	4.08 – 4.68	4.01 – 4.20
Echo Time (ms)	3.13	3.13	3.13	1.79 – 2.09	1.73 – 1.82
Flip Angle (°)	15	15	13 - 15	13	13
Fat-Saturated	Yes	Yes	Yes	Yes	Yes
Number of Averages	0.7	0.7	0.7	0.7	0.7
Temporal Resolution (s)	10 - 12	12 - 15	7 - 15	6 - 10	7 - 11
Reconstructed Matrix	256 × 256	256 × 256	256 × 256	256 × 256	256 × 256
Reconstructed Resolution	0.94 × 0.94	0.94 × 0.94	0.94 x 0.94	0.94 × 0.94	0.94 × 0.94
Slice Thickness (mm)	3.0	3.0	3.0	3.0	3.0
Slice Gap (mm)	0.0	0.0	0.0	0.0	0.0
Field of View (cm^2)	24	24	24	24	24

Table S1: Summary table of sequence parameters for **A**. axial T2-weighted imaging (T2WI), **B**. axial diffusion-weighted imaging (DWI) and **C**. axial dynamic contrast-enhanced (DCE) MRI. I. and II. in B indicate two diffusion protocols for acquired and calculated diffusion b values. ***Abbreviations:*** *SE = Spin-Echo, GR = Gradient-Echo, EP = Echo-Planar, PROP = Propeller.*

.

PSAd Cut-off	Comparison	Metric	(95% CI)	P-value
0.12	≤40 mL vs 40–60 mL	Sensitivity	(0.14, 0.29)	<0.001
		Specificity	(–0.31, –0.17)	<0.001
	≤40 mL vs ≥60 mL	Sensitivity	(0.44, 0.63)	<0.001
		Specificity	(–0.42, –0.30)	<0.001
	40–60 mL vs ≥60 mL	Sensitivity	(0.21, 0.43)	<0.001
		Specificity	(–0.17, –0.08)	<0.001
0.15	≤40 mL vs 40–60 mL	Sensitivity	(0.16, 0.33)	<0.001
		Specificity	(–0.23, –0.11)	<0.001
	≤40 mL vs ≥60 mL	Sensitivity	(0.48, 0.66)	<0.001
		Specificity	(–0.28 to –0.17)	<0.001
	40–60 mL vs ≥60 mL	Sensitivity	(0.22, 0.43)	<0.001
		Specificity	(–0.09, –0.03)	<0.001
0.20	≤40 mL vs 40–60 mL	Sensitivity	(0.14, 0.33)	<0.001
		Specificity	(–0.17, –0.09)	<0.001
	≤40 mL vs ≥60 mL	Sensitivity	(0.36, 0.53)	<0.001
		Specificity	(–0.19, –0.10)	<0.001
	40–60 mL vs ≥60 mL	Sensitivity	(0.11, 0.31)	<0.001
		Specificity	(–0.034, 0.001)	0.033

Table S2: Pairwise comparisons of sensitivity and specificity for detecting clinically significant prostate cancer (csPCa) using PSA density thresholds of 0.12, 0.15, and 0.20 ng/mL², stratified by prostate volume groups (≤40 mL, 40–60 mL, and ≥60 mL)

.
